# DEMENTIA AND THE ROLE OF EVIDENCE-BASED GUIDELINES IN PRIMARY CARE: RESULTS OF A CLUSTER RANDOMIZED CONTROLLED TRIAL

**DOI:** 10.1093/geroni/igac059.1829

**Published:** 2022-12-20

**Authors:** Sonia Lech, Julie L O'Sullivan, Robert P Spang, Jan-Niklas Voigt-Antons, Paul Gellert, Johanna Nordheim

**Affiliations:** Charité – Universitätsmedizin Berlin, Berlin, Berlin, Germany; Charité-Universitätsmedizin Berlin, Berlin, Berlin, Germany; Technische Universität Berlin, Berlin, Berlin, Germany; University of Applied Sciences Hamm-Lippstadt, Hamm, Nordrhein-Westfalen, Germany; Charité – Universitätsmedizin Berlin, Berlin, Berlin, Germany; Charité – Universitätsmedizin Berlin, Berlin, Berlin, Germany

## Abstract

General practitioners (GPs) play a key role in the care of people with dementia (PwD). However, the potentials of evidence-based dementia guidelines in primary care remain unclear. The main objective of the present study was to examine the role of guideline-based dementia care in general practices and to evaluate the effectiveness of a tablet-based intervention aiming at improving guideline-based care. A two arm, cluster-randomized controlled trial (cRCT) with an intervention group (tablet-based intervention) and a control group (treatment as usual) was conducted from April 15, 2018, to July 31, 2021, in Berlin and the surrounding area. Primary outcome was defined as adherence to dementia guideline recommendations after 9 months. Secondary outcomes included various health outcomes assessed in PwD (e.g., quality of life). Linear Mixed Models were used to analyse cross sectional baseline data. Primary and secondary outcomes were analysed by intention-to-treat analysis and using Mixed Models. Data of N = 28 GPs and N = 91 PwD was analysed. PwD were on average 80.5 years old (SD = 6.3), 59% were female. Self-reported overall guideline adherence of GPs was on average 71% (SD = 19.4, range: 25–100). Further, lower guideline adherence was found to be significantly associated with higher numbers of patients (γ10 = − 5.58, CI = − 10.97, − 0.19, p = .04). The effectiveness of the cRCT is still under evaluation. Final results will be presented and discussed at the GSA Annual Scientific Meeting. Further, implications and conclusions of the trial will be provided.

